# Trabecular Juvenile Ossifying Fibroma Involving the Maxilla: A Rare Case

**DOI:** 10.7759/cureus.71183

**Published:** 2024-10-10

**Authors:** Deeksheetha Prabhu Venkatesh, Karthikeyan Ramalingam, Pratibha Ramani, Gheena S, Murugesan Krishnan

**Affiliations:** 1 Oral Pathology and Microbiology, Saveetha Dental College and Hospitals, Saveetha Institute of Medical and Technical Sciences, Saveetha University, Chennai, IND; 2 Oral and Maxillofacial Surgery, Saveetha Dental College and Hospitals, Saveetha Institute of Medical and Technical Sciences, Saveetha University, Chennai, IND

**Keywords:** exophthalmos, facial asymmetry, female, fibro osseous lesion, juvenile ossifying fibroma, juvenile trabecular ossifying fibroma, maxilla, ossifying fibroma, osteolytic lesion, trabecular juvenile ossifying fibroma

## Abstract

Trabecular juvenile ossifying fibroma (TrJOF) is a rare, benign, fibro-osseous lesion that predominantly affects children and adolescents. The lesion is locally aggressive, has a high recurrence rate, and is often misdiagnosed due to its clinical and radiographic similarities to other lesions. A seven-year-old female presented with a history of swelling and pain on the right side of her face for the last month. Extraoral examination revealed facial asymmetry and exophthalmos of the right eye. Intraoral examination showed obliteration of the buccal vestibule, though the mucosal lining and teeth were intact. Cone-beam computed tomography (CBCT) demonstrated a large, expansile, osteolytic lesion occupying the right maxillary sinus and adjacent structures, suggesting a fibro-osseous lesion. The enucleated specimen showed a highly cellular connective tissue stroma with irregular bony trabeculae rimmed by osteoblasts, numerous multinucleated giant cells, and basophilic cementum-like calcifications. The histopathological evaluation confirmed the diagnosis of trabecular juvenile ossifying fibroma. This case highlights the diagnostic challenges of TrJOF due to its overlapping features with other fibro-osseous and giant cell lesions. Early diagnosis and surgical intervention are critical in preventing local destruction and recurrence.

## Introduction

Fibro-osseous lesions (FOLs) of the jaw are a diverse group of disorders characterized by the replacement of normal bone with a benign proliferation of fibrous tissue and newly formed mineralized material [1]. These lesions encompass a spectrum of diseases, including fibrous dysplasia, ossifying fibroma, and cemento-osseous dysplasia, and there is significant overlap in their histopathological presentations, making diagnosis an arduous task. FOLs primarily affect the craniofacial bones, often leading to functional and aesthetic concerns due to their expansive nature [2]. 

Ossifying fibroma (OF) is a well-demarcated, sometimes encapsulated benign fibro-osseous lesion that commonly affects women more than men with a predisposition to be diagnosed in the second and third decades of the patient's life [3]. Kannan et al. [4] reported 23 cases of OF over three years in their study. It is characterized by the substitution of normal bone by fibrous connective stroma along with varied amounts of newly formed unmineralized bone and sometimes along with cementum-like calcifications [5]. It is most often encountered in the mandible, with a predominance in the molar and premolar regions, but may also occur in the maxilla and other craniofacial bones [6]. 

Ossifying fibroma can be further subdivided into two histopathological variants: conventional ossifying fibroma, which is prevalent in adults, and juvenile ossifying fibroma (JOF), a rarer, more aggressive form seen predominantly in children and young adults [5]. JOF is particularly significant due to its rapid growth, potential for recurrence, and frequent occurrence in the maxillofacial region. Understanding the clinical course and management of JOF is essential for minimizing morbidity and ensuring optimal patient outcomes [6]. 

JOF is distinct from the conventional OF due to its earlier onset, more aggressive behavior, and higher recurrence rate [6]. It is further subdivided into two histopathological variants: trabecular juvenile ossifying fibroma (TrJOF) and psammomatoid juvenile ossifying fibroma (PsJOF), which have unique histopathological features. TrJOF is characterized by trabeculae of woven bone, and PsJOF shows small, round ossicles resembling psammoma bodies [7]. We discuss such a rare presentation of TrJOF in this case report.

## Case presentation

A seven-year-old girl was referred to Saveetha Dental College and Hospitals, Chennai, India, for the management of facial swelling. The patient had undergone an incisional biopsy in another institution and was diagnosed with central giant cell granuloma. The patient had reported to our institution for further management. 

Her chief complaint was pain and swelling on the right side of her face for the past month. The patient’s history revealed that the pain was non-spontaneous, primarily aggravated by pressure applied to the swelling, and resolved spontaneously without intervention. There was no history of paresthesia, visual disturbances, or other neurological symptoms. Informed consent was obtained from the patient’s parents for further examination, diagnosis, and treatment. The patient’s medical history was non-contributory, and there were no significant alterations in their overall health status.

**Figure 1:**
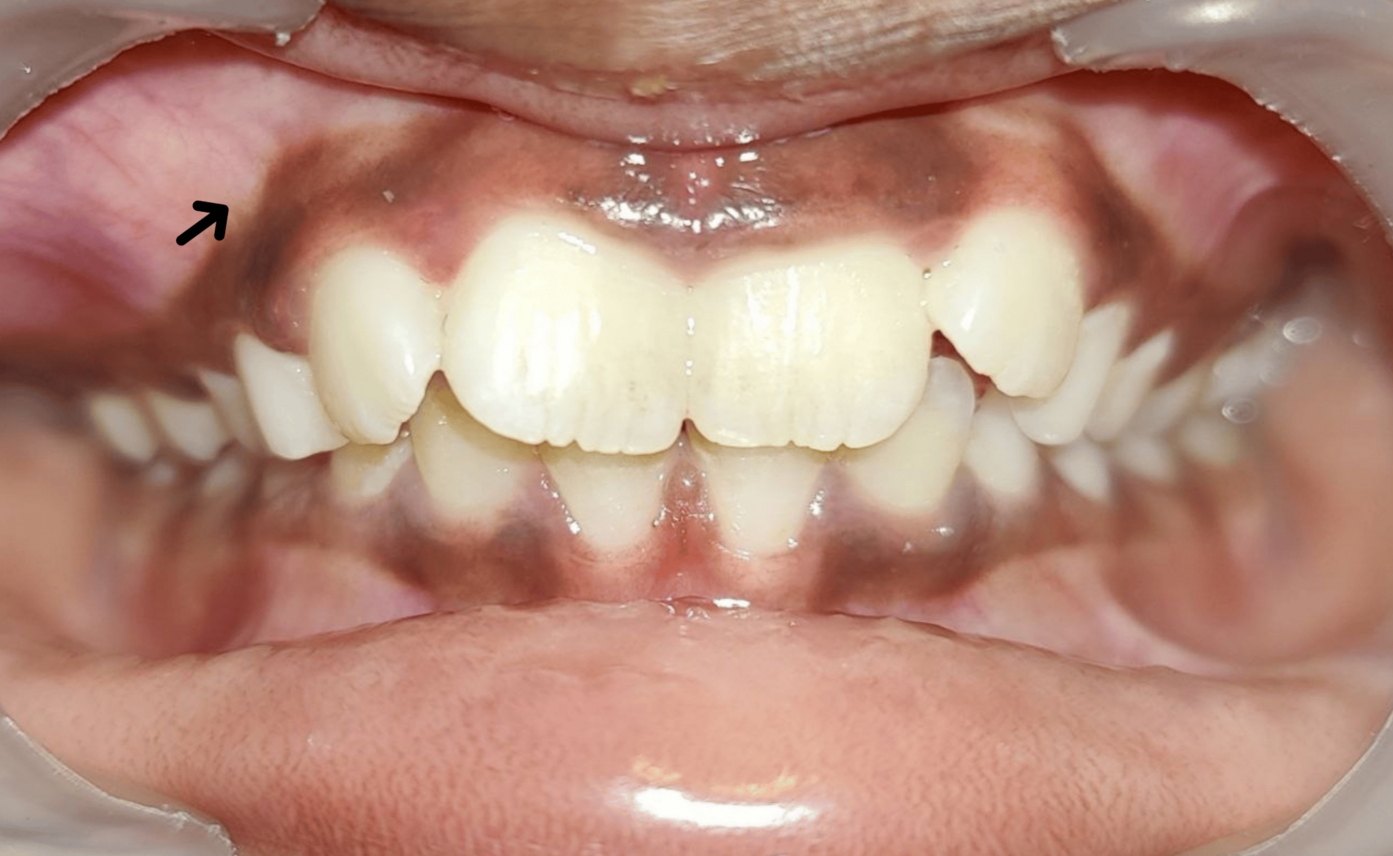
Clinical picture Intraoral frontal view showing obliteration of buccal vestibule on the right side (black arrow)

An extraoral examination of the patient revealed a prominent facial asymmetry with a noticeable swelling on the right side of the face. The patient also reported mild pain that had developed one month before the consultation. On physical examination, a distinct swelling was observed over the right cheek region, accompanied by exophthalmos of the right eye, suggesting potential orbital involvement. Intraoral examination revealed obliteration of the buccal vestibule on the right side (Figure 1). The mucosal lining appeared intact with no signs of inflammation. Additionally, the teeth in the affected area were clinically sound, with no mobility or signs of pathological involvement. Notably, there was no evidence of cervical or submandibular lymphadenopathy, and the general examination did not reveal any systemic involvement.

**Figure 2:**
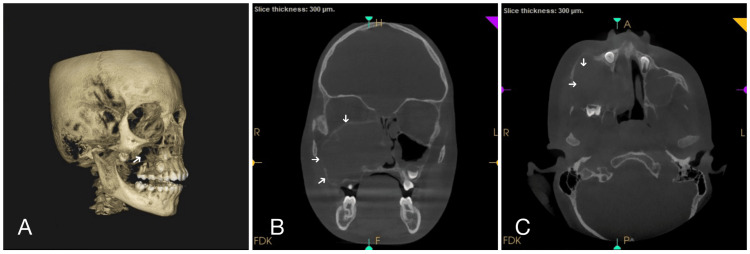
Computed tomography A: CBCT reveals the destruction of cortical bone in relation to 53, 54, 55, and 16 with involvement of infraorbital bone; 2B: CBCT shows an expansile, osteolytic lesion occupying the right maxillary sinus with extension into adjacent structures, and the lesion is seen involving the right side of the nasal cavity and the nasal septum; 2C: CBCT reveals an osteolytic lesion involving the maxilla with cortical bone perforation. CBCT: cone-beam computed tomography

Cone-beam computed tomography (CBCT) revealed a large, expansile, osteolytic lesion occupying the right maxillary sinus, extending into the adjacent structures. The lesion involved the right ethmoid air cells, the anterior wall of the sphenoid sinus, and the nasal cavity, particularly affecting the nasal septum. Additionally, the involvement of the floor and medial wall of the right orbit contributed to the observed exophthalmos. The lesion also caused the distal displacement of a developing tooth. The radiographic features, including the expansile and osteolytic characteristics, suggested a fibro-osseous lesion, though differential diagnoses such as an aggressive neoplasm or granulomatous lesion were considered (Figure 2).

A previous incisional biopsy performed at another institution led to a diagnosis of central giant cell granuloma. This raises the possibility of a misdiagnosis or an evolving lesion, warranting further histopathological evaluation to confirm the nature of the lesion.

**Figure 3:**
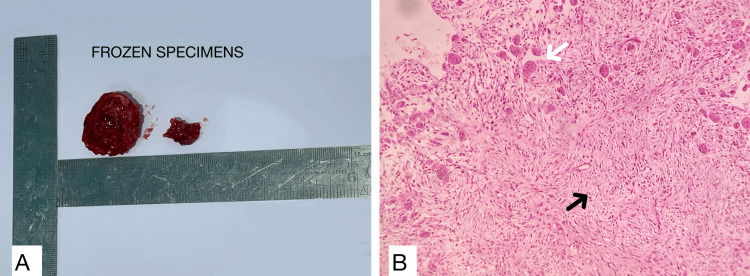
Intraoperative diagnosis A: image showing two hard tissue specimens submitted for the frozen section; B: photomicrograph of H&E stained frozen sections (10x) that show whorling of spindle cells (black arrow)with numerous multinucleated giant cells (white arrow).

During the frozen section, two hard tissue bits were received (Figure 3A). The soft tissue was scraped for histopathological frozen examination, which revealed a highly cellular stroma with spindle-shaped cells and osteoclasts. The spindle-shaped cells exhibited a swirling pattern along with osteoclasts and a few bony spicules with osteoblastic rimming (Figure 3B). The intraoperative diagnoses were juvenile cementoossifying fibroma with aneurysmal bone cyst-like areas or central giant cell granuloma.

**Figure 4:**
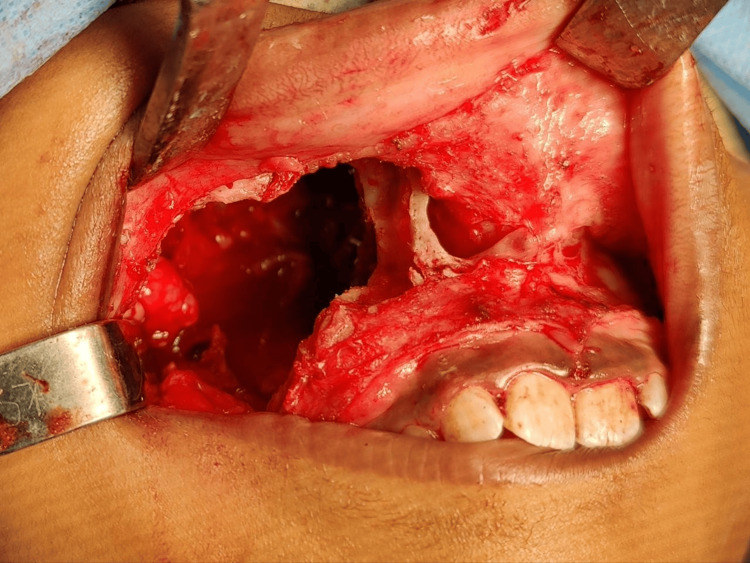
Intraoperative picture

Surgical excision was planned under general anesthesia. A crevicular incision was placed from the 16-63 region. A full-thickness mucoperiosteal flap was elevated to disclose the lesion. Bone guttering was done, and a bone window was created. Complete excision and curettage were followed by debridement with surgical dressing. The wound was closed with Vicryl resorbable sutures after achieving hemostasis. Post-operative medication, including analgesics and antibiotics, was given with instructions on a soft diet and an extra-oral ice pack application for five days (Figure 4).

**Figure 5:**
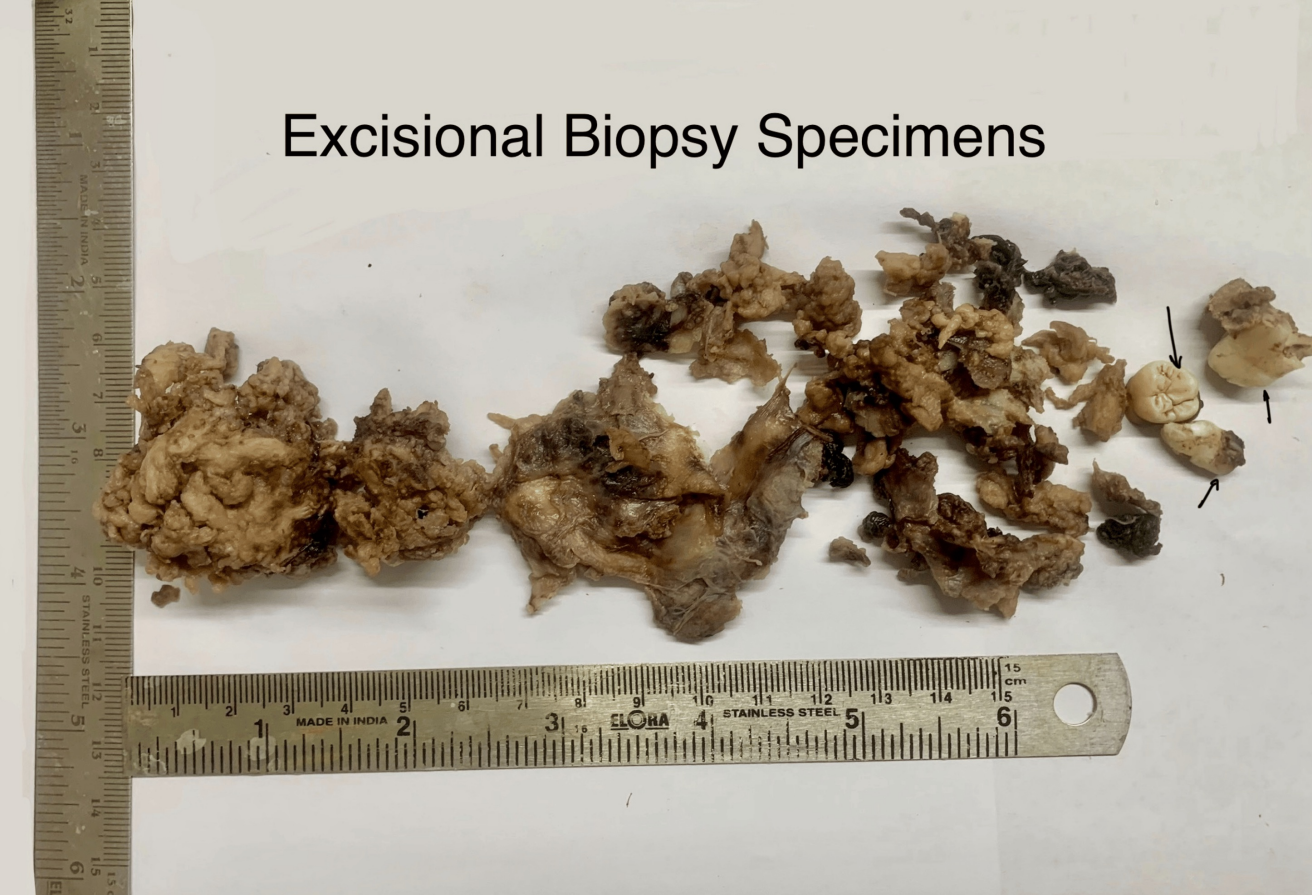
Excisional biopsy specimen Gross image of the excisional biopsy specimen with soft tissue and hard tissue containing bone bits and teeth (53, 54, 55, indicated by black arrow marks).

The excised specimen was received as multiple hard and soft tissue bits received in formalin, brownish yellow, the largest bit measuring 3.9 x 3.0 x 1.5 cm. Soft tissue specimens were kept for routine processing, and hard tissue was kept for decalcification under 40% formic acid (Figure 5).

**Figure 6:**
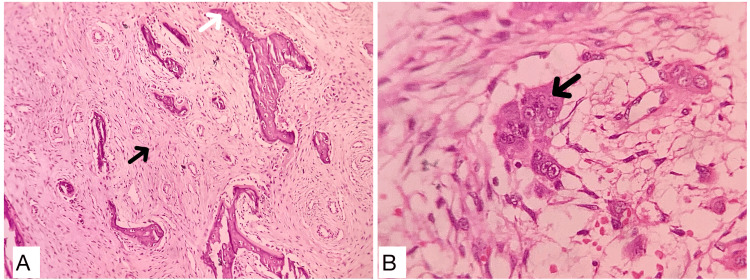
Histopathological photomicrographs A: H&E-stained section (10x) showing cellular stroma with numerous spindle-shaped cells (black arrow) along with areas of osteoid formation (white arrow); B: H&E-stained section (40x) shows multinucleated giant cells (black arrow) in a fibrocellular stroma.

Histopathological sections revealed a highly cellular connective tissue stroma, characterized by numerous spindle-shaped cells with scant cytoplasm and basophilic nuclei, arranged in a whorling pattern. Areas of osteoid formation were noted, along with irregular bony trabeculae rimmed by osteoblasts. The presence of numerous multinucleated giant cells, containing 6-12 nuclei, suggested osteoclastic activity. Additionally, basophilic cementum-like calcifications were evident throughout the stroma. In deeper planes, mature fibrous connective tissue with moderate vascularity, peripheral bone, and areas of hemorrhage were observed (Figure 6). Histopathologically, it was suggestive of trabecular juvenile ossifying fibroma (TrJOF). 

**Figure 7:**
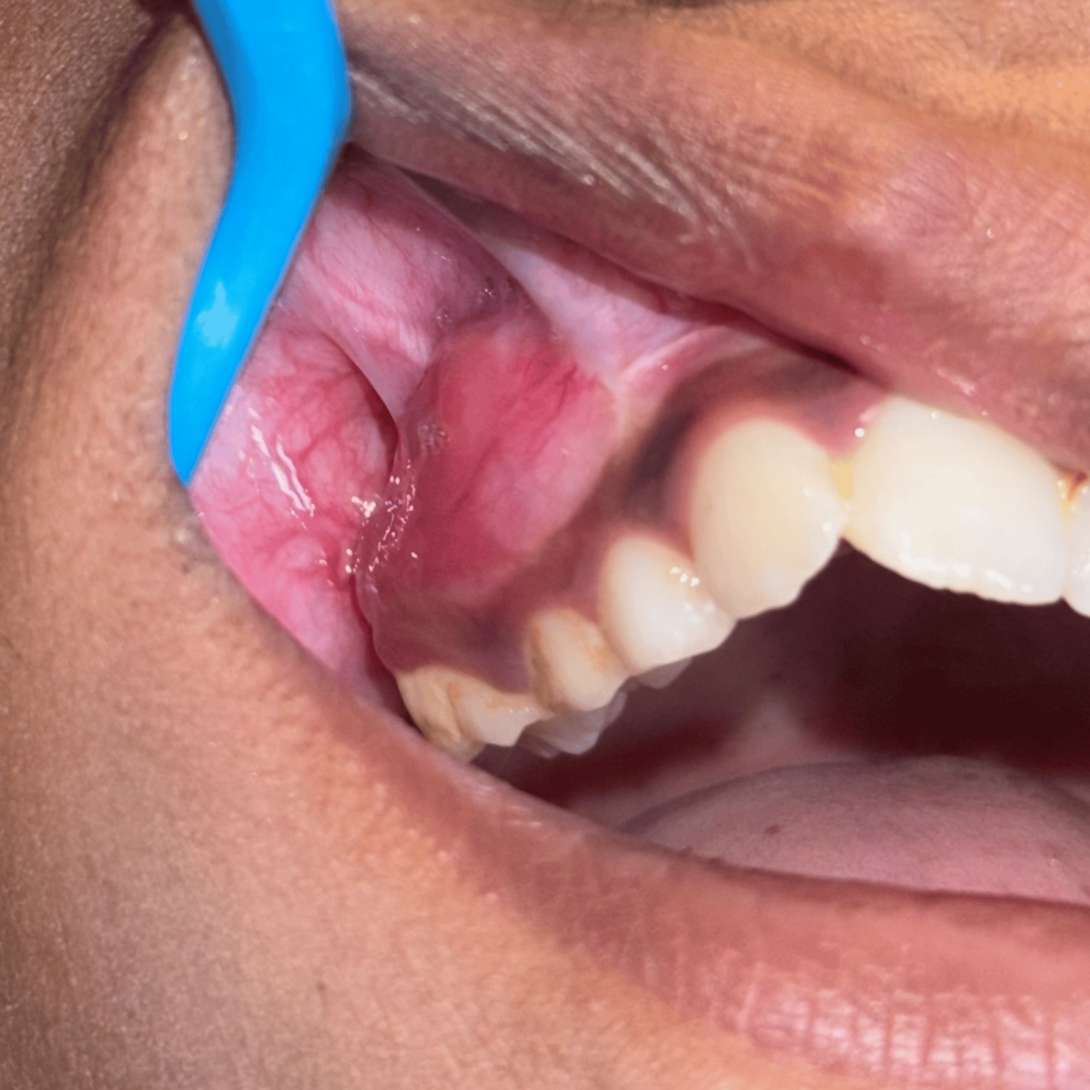
Follow-up picture

The patient remains disease-free on a 16-month follow-up (Figure 7). She is instructed to maintain good oral hygiene and make periodic visits. Orthodontic treatment needs to be evaluated after eruption of permanent teeth.

## Discussion

JOFs are a rare, benign fibro-osseous lesion, typically affecting young individuals. JOF is distinct from other ossifying fibromas due to its aggressive clinical behavior and tendency for recurrence. The incidence of JOF is difficult to ascertain due to its rarity, but it is generally considered an uncommon lesion, representing approximately 2% of all fibro-osseous lesions of the jaws [8]. A Pubmed search (Juvenile Ossifying Fibroma [AND] Jaws) yielded 88 articles on JOF, indicating their uncommon nature. A literature search has revealed 31 cases of the trabecular variant and 29 cases of the psammatoid variant of JOF. However, its aggressive growth pattern and potential for recurrence make it a significant clinical entity. Studies have reported recurrence rates as high as 30-58% following surgical resection, highlighting the need for careful long-term follow-up [8].

Histopathological examination is crucial for accurate diagnosis, with TrJOF and PsJOF each exhibiting distinct microscopic features. The management of JOF primarily involves surgical excision, with enucleation and curettage being the preferred approaches for small, well-circumscribed lesions, while more extensive resections may be necessary for larger, aggressive lesions [9]. Due to the high recurrence rates, close postoperative monitoring is recommended. In the present case, the lesion predominantly affected the maxillary sinus with orbital involvement, leading to exophthalmos. Similar literature reports that orbital involvement often results in proptosis, as observed in studies by Han et al. [10]. These cases underscore the aggressive nature of JOF, especially when arising in anatomically sensitive areas such as the orbit and maxillofacial region.

The clinical progression of JOF, characterized by a slow-growing but aggressive nature, aligns with findings from previous reports. El-Mofty highlighted the aggressive potential of JOF, noting frequent recurrence after incomplete excision [11]. This aggressiveness is especially true for PsJOF, which is associated with more extensive involvement and a higher recurrence rate than TrJOF. The case presented here demonstrates a similar pattern, where the lesion extended into multiple adjacent structures, including the maxillary sinus and the nasal cavity, consistent with a report by Osunde et al. [12], who documented cases of JOF with widespread extension. However, despite its aggressive clinical course, JOF remains a benign lesion. This contrasts with malignant lesions like osteosarcoma, which exhibit more destructive behavior and metastasis [13]. The absence of paresthesia, visual disturbances, or systemic symptoms in this case further supports the benign nature of the lesion [6]. 

Histologically, the current case displayed the classic features of TrJOF, with spindle-shaped cells in a whorling pattern, osteoid formation, and multinucleated giant cells. These findings are in line with those described by Slootweg and Panders [2], who emphasized the cellular nature of JOF with abundant osteoid and the presence of multinucleated giant cells. These cellular features are seen in both variants of JOF but are more pronounced in the trabecular type, where the stroma is densely packed with fibroblasts arranged in a whorling pattern [11]. Additionally, psammomatous calcifications further support the diagnosis of PsJOF, as these structures are a hallmark of the psammomatoid variant, as described by Eversole et al. [1]. Our present case showed moderate vascularity and areas of hemorrhage similar to Beheret et al. [13], wherein the lesion displayed a richly vascular stroma with areas of hemorrhage. This vascular nature contributes to the lesion’s rapid growth and expansile behavior. The histopathological differentiation between TrJOF and PsJOF is crucial for prognosis, as the trabecular variant tends to show a lower recurrence rate than its psammomatoid counterpart [11], which found a significant correlation between the histological variant and clinical outcome. The patient's old biopsy report of central giant cell granuloma (CGCG) illustrates the challenges in differentiating JOF from other giant cell lesions. CGCG often shares radiographic and histological features with JOF, including multinucleated giant cells and a fibrocellular stroma. However, cementum-like calcifications and osteoid formation in the current case point more toward JOF, further highlighting the need for careful histopathological examination [14]. 

In the study by Omar Solyman [15], a four-year-old male patient with autism presented with left progressive proptosis for three weeks. Radiographically, an expansile lesion involving the left maxillary sinus with areas of hyper and hypodensities with a hyperdense border was noted. Histopathologically, the lesion showed hyper- and hypocellular areas with areas of osteoid formation along with scattered multinucleated giant cells. These findings were similar to our present case, which showed an expansile osteolytic lesion that showed cortical destruction. Histopathologically, our case was hypercellular with numerous osteoclastic giant cells. The management of JOF typically involves surgical excision, with the extent of surgery depending on the size and location of the lesion. As demonstrated by Eversole et al. [1], and by previous cases of OF [3,16], central giant cell granuloma [17], and cementoosseous dysplasia [18], complete excision with clear margins is the treatment of choice, which is similar to the treatment for JOF. In the present case, despite the high recurrence rate of JOF, particularly the psammomatoid variant, due to the orbital floor involvement, surgical management is complicated as an aggressive resection may result in functional or cosmetic defects; hence, enucleation was carried out. Recurrence remains a significant concern, with previous studies [9] reporting recurrence rates as high as 30-50% for PsJOF. This underscores the need for long-term follow-up in cases involving incomplete excision or where radical surgery is not feasible due to anatomical constraints.

Clinical relevance

Our case falls within the typical age range for TJOF, which predominantly affects children and young adults. The one-month history of facial swelling, pain, and associated exophthalmos indicates the lesion’s rapid growth, a hallmark of aggressive juvenile ossifying fibromas. Both central giant cell granuloma and TJOF can share overlapping histological features, such as multinucleated giant cells, complicating the initial diagnosis. However, precise differentiation between these entities is critical, as TJOF demands more aggressive surgical management due to its rapid expansion and osteolytic behavior.

Due to the high recurrence rate associated with TJOF, particularly if the lesion is not completely excised, long-term follow-up is essential to monitor for regrowth. This case emphasizes the importance of early recognition and intervention to prevent serious complications, including facial deformity, functional impairment, and further extension into vital areas such as the orbit. The aggressive nature of TJOF underscores the need for timely and comprehensive treatment to ensure optimal outcomes.

## Conclusions

This case underscores the aggressive behavior of juvenile ossifying fibroma (JOF), particularly when it arises in the maxillofacial region. The clinical, radiographic, and histopathological findings in this case align with previously documented cases in the literature, further highlighting the challenges in diagnosis and management. A multidisciplinary approach, combining thorough radiographic assessment, careful histopathological examination, and appropriate surgical intervention, remains critical in the successful treatment of JOF. Continued follow-up is essential due to the risk of recurrence. 
